# Bilayer Type I Atelocollagen Scaffolds for In Vivo Regeneration of Articular Cartilage Defects

**DOI:** 10.3390/bioengineering12050446

**Published:** 2025-04-24

**Authors:** Sang Hun Woo, Bo Keun Lee, Andrew S. Kwak, Jin Hyo Yang, Seo Yeon Kim, Man Soo Kim, Ji Chul Yoo

**Affiliations:** 1 Research & Development Headquarters, Cellontech Co., Ltd., Seoul 04783, Republic of Korea; wsh@cellontech.com (S.H.W.);; 2 Department of Orthopaedic Surgery, College of Medicine, Seoul St. Mary’s Hospital, The Catholic University, Seoul 06591, Republic of Korea

**Keywords:** articular cartilage, extracellular matrix, microfracture, regeneration, collagen

## Abstract

Articular cartilage has limited regenerative potential due to its anatomical characteristics, making complete recovery from damage challenging. Microfracture (MFx) is a widely used technique to promote cartilage healing, often enhanced with scaffolds to improve outcomes. In this study, we compared the efficacy of bilayer atelocollagen and standard collagen scaffolds combined with MFx in treating osteochondral defects in a rabbit model. Three articular cartilage defects were created in the femoral condyle of each rabbit and treated with either MFx plus a bilayer atelocollagen scaffold (test group), MFx plus a standard collagen scaffold (positive group), or MFx alone (negative group). Macroscopic and histological assessments were performed at 3, 6, and 12 weeks. By week 12, macroscopic examination showed hyaline-like cartilage restoration in the test group, while the positive group exhibited restoration with some overgrowth, and the negative group showed no restoration. Histological analysis revealed significantly better restoration in the test group than in the negative group, with comparable outcomes between the test and positive groups. These findings suggest that bilayer atelocollagen scaffold implantation following MFx is a promising treatment for articular cartilage defects and may provide a viable therapeutic option for patients with cartilage damage.

## 1. Introduction

Articular cartilage is a highly specialized tissue composed of chondrocytes, some progenitor cells, and an abundant extracellular matrix (ECM). The ECM of articular cartilage is primarily composed of a collagen and proteoglycan network, which provides both structural integrity and mechanical resilience. Chondrocytes within articular cartilage reside, proliferate, and differentiate inside the supportive environment [1]. However, adult cartilage has low metabolic activity and limited regenerative capacity. Its high stiffness and lack of vascularity restrict cell migration, while its histological structure prevents chondrocytes from forming large cell clusters, leading to a shortage of essential cells. Additionally, collagen limits progenitor cell recruitment at both the articular surface and synovial lining, further hindering chondral defect repair [2]. Microfracture (MFx), osteochondral autologous transplantation, and autologous chondrocyte implantation are among the most effective treatments for cartilage injuries [3]. MFx, in particular, is widely used for localized cartilage defects and has demonstrated promising outcomes in both mid- and long-term follow-up studies [4,5].

MFx stimulates bone marrow by inducing localized bleeding to promote cartilage repair [6,7]. This is achieved by creating multiple perforations in the subchondral bone plate using the sharp tip of a microfracture awl, allowing mesenchymal stem cells (MSCs) to migrate from the bone marrow cavity to the cartilage lesion [8]. However, MFx has several limitations. First, the fibrin clot that forms at the injury site has poor mechanical stability, is vulnerable to tangential forces [9], and can be easily washed away by synovial fluid. Second, the repair tissue formed after MFx is prone to damage from axial forces, potentially compromising chondrocyte regenerative capacity. Additionally, the regenerated tissue often consists of fibrocartilage rather than true hyaline cartilage. Fibrocartilage, rich in type I collagen and low in proteoglycans, is less resistant to wear and tear. Consequently, while 60–70% of patients experience symptom relief for up to 2 years post-surgery, structural degradation often leads to worsening symptoms, particularly in larger defects [10,11,12,13]. Recently, enhanced MFx techniques have been developed to improve chondrogenesis and support more effective cartilage regeneration [14,15,16].

Collagen is a key component of the ECM in human tissues and plays a crucial role in tissue and organ development due to its biocompatibility [17]. Collagen-based scaffolds are widely used in cartilage regeneration as they provide structural support and closely mimic the native ECM. Implanting a collagen matrix scaffold enhances mechanical stability while creating a favorable environment for chondrogenic differentiation and cartilage regeneration [18]. The efficacy of collagen scaffolds has been demonstrated in both in vitro and in vivo cartilage regeneration studies [19], with therapeutic effects evaluated based on their physicochemical properties and surface characteristics [20]. However, direct collagen implantation may trigger an immune response, as the body can recognize it as an antigen. To mitigate this, telopeptide-removed atelocollagen is used to minimize immunogenicity [21,22]. This low-antigenicity atelocollagen is widely applied in cartilage tissue regeneration research. In previous studies, the effectiveness of monolayer atelocollagen scaffolds in microfracture techniques and autologous bone marrow concentrate procedures has been evaluated for the joint defect, and the treatment result of monolayer atelocollagen scaffold was better in either Mfx and bone marrow aspirate concentrate procedure compared to other collagen or the no collagen group [23].

In this study, we developed a bilayer atelocollagen scaffold and validated its efficacy in osteochondral defect repair when combined with the MFx technique. Bilayer atelocollagen scaffold is composed of two structurally different collagen layers to support regeneration by providing tissue-specific biological substrates. We hypothesized that implanting a bilayer atelocollagen scaffold would enhance the healing process and compared its effectiveness with other collagen scaffolds.

## 2. Materials and Methods

### 2.1. Experimental Design

**Figure 1:**
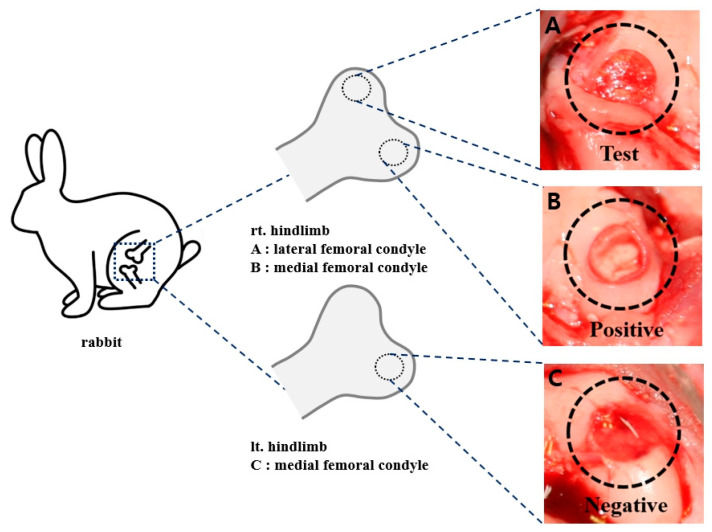
Schematic illustration of the experimental surgical design. Rt. hindlimb lateral femoral condyle (**A**: Test group), Rt. hindlimb medial femoral condyle (**B**: Positive group), Lt. hindlimb medial femoral condyle (**C**: Negative group).

In this study, we aimed to compare the efficacy of bilayer atelocollagen and standard collagen scaffolds when used in combination with MFx for treating osteochondral damage in a rabbit model. Male New Zealand White rabbits (Saeronbio, Gyeonggi-do, Republic of Korea) were used. Three articular cartilage defects were created in the femoral condyle of each rabbit, with each artificially created defect receiving one of the following treatments: MFx with a bilayer atelocollagen scaffold (test group), MFx with a collagen scaffold (positive group), or MFx alone (negative group) (Figure 1).

### 2.2. Preparation of the Atelocollagen and Collagen Scaffolds

The atelocollagen used in this study is collagen from which telopeptides have been removed using enzymatic treatment. This process retains the same characteristics as native collagen while reducing antigenicity, resulting in a lower inflammatory response. Bilayer type I atelocollagen scaffolds (RegenPatch) were produced by Cellontech Co., Ltd. (Seoul, Republic of Korea). Type I/III collagen scaffolds (Chondro-Gide) were produced by Geistlich Pharma AG Co., Ltd. (Wolhusen, Switzerland).

### 2.3. Animal Experiments

All animal experiments were conducted in accordance with the guidelines of the Animal Care and Use Committee [24].

Twelve male New Zealand White rabbits (25 weeks old, 3.5–4.0 kg) were housed in standard rabbit cages (420W × 500D × 350H mm) under controlled conditions: a 12-h light-dark cycle, room temperature of 22 ± 3 °C, and humidity of 50 ± 10%. The rabbits were acclimated for 4 weeks before the study and had access to standard laboratory food pellets and water ad libitum. Following surgery, all rabbits were allowed free movement in their cages without splints. The animals were sacrificed using a carbon dioxide euthanasia chamber at 3, 6, and 12 weeks following the operation. The entire knee was dissected, macroscopically examined, and photographed. The distal femurs were fixed in 10% buffered formalin (Sigma-Aldrich, Saint Louis, MO, USA). Cartilage regeneration was assessed histologically using hematoxylin-eosin and Safranin O staining.

Immunohistochemistry was performed with antibodies against Type I and Type II collagen following standard immunohistochemical protocols. Histological analysis was conducted using a modified semi-quantitative grading system.

### 2.4. Surgical Procedure

All surgeries were conducted in a sterile environment. Anesthesia was induced via intramuscular injection of a Zoletil (Verbac, France) and Rompun (Bayer, Germany) mixture, adjusted according to the animal’s weight. A medial parapatellar incision was made to open the joint, and the patella was dislocated with the leg in full extension. A full-thickness defect (4 mm in diameter, 3 mm in depth) was created in the articular cartilage and subchondral bone of the femoral condyle using an electric burr. MFx was then performed on each defect. The arthrotomies were closed in layers using 3.0 Vicryl sutures (Ethicon Inc., Somerville, NJ, USA) for the deep layer and 3.0 nylon sutures (Ailee, Busan, Republic of Korea) for the skin. Postoperatively, the animals were divided into three groups: Test group: implanted with bilayer atelocollagen scaffolds; Positive group: implanted with collagen scaffolds; and Negative group: no additional treatment. Fibrin glue (GreenPlast, Green Cross, Republic of Korea) was applied to all implant sites for fixation. Postoperative care included daily intramuscular injections of Cefazolin (Chong Kun Dang, Seoul, Republic of Korea) for 3 days and daily disinfection of surgical wounds with povidone-iodine solution (Samil, Seoul, Republic of Korea). Skin sutures were removed 7 days after surgery.

### 2.5. Macroscopic Analysis

Following euthanasia, the distal femurs were harvested at 3, 6, and 12 weeks. A macroscopic assessment of the knee joint was performed to evaluate the repair’s surface properties, its integration with nearby host tissue, and any osteoarthritic changes. The graft surfaces were carefully examined for color, structural integrity, contour, and smoothness.

### 2.6. Histological Analysis

The collected tissues were fixed, decalcified, dehydrated, cleared, and embedded in paraffin. Serial sections (5 µm thick) were prepared using a microtome (Thermo Shandon, Finesse ME Microtome, Uppsala, Sweden) and stained with hematoxylin–eosin (KPNT, SHE-001, Republic of Korea) to assess general tissue morphology. To evaluate cartilage matrix composition, sections were stained with Safranin O (Scytek Laboratories, SOC-1, Utah, USA) to assess negatively charged matrix proteoglycans and with toluidine blue (Sigma-Aldrich, Saint Louis, MO, USA) to visualize glycosaminoglycan distribution. Immunohistochemistry for Type I and Type II collagen was performed using a polymer-based protocol (Vector Laboratories, Burlingame, CA, USA). The following primary antibodies were used: Type I collagen (Abcam, Cambridge, ab6308, UK) and Type II collagen (Merck, CP-18, Darmstadt, Germany).

Histological assessment was performed using a modified scoring system based on O’Driscoll, Pineda, and Wakitani [25,26,27,28]. The scoring system consists of seven categories, with scores ranging from 0 to 23 points (Table 1), where a lower score signifies improved cartilage repair.

### 2.7. Statistical Analyses

Data are expressed as means ± standard deviations. Statistical comparisons were performed using a two-way analysis of variance (ANOVA) in Microsoft Excel (Microsoft Corp., Redmond, WA, USA, version 2019). A *p*-value of <0.05 was considered statistically significant.

## 3. Results

### 3.1. Macroscopic Observation

**Figure 2:**
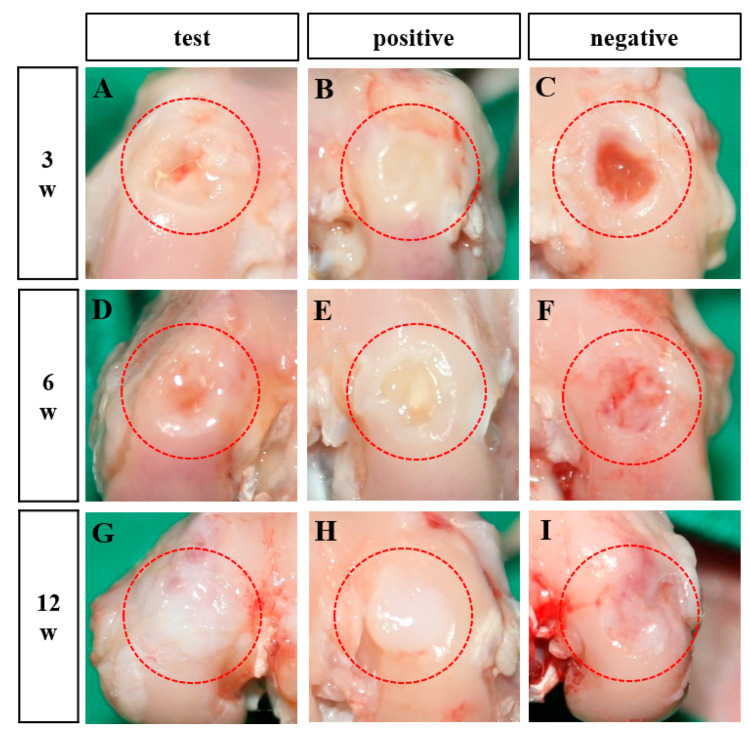
Macroscopic evaluation of artificial defects along the femoral condyle of the distal femur at 3 weeks (**A**–**C**), 6 weeks (**D**–**F**), and 12 weeks (**G**–**I**) after surgery. Red dotted lines indicate defect sites (n = 4). (**A**,**D**,**G**): Bilayer atelocollagen scaffold-implanted test group; (**B**,**E**,**H**): Collagen scaffold-implanted positive group; (**C**,**F**,**I**): Non-treated negative group.

Following surgery, the rabbits exhibited no adverse effects, such as weight loss, inflammation, or mortality. The regenerated cartilage was visually assessed for smoothness, color, and structural integrity. Figure 2 shows the condition of the defect sites at 3-, 6-, and 12-weeks post-treatment. At 3 weeks, the defect sites in the test and negative groups remained incompletely filled, while the positive group exhibited partial filling with irregular fibrous tissue (Figure 2A–C). At 6 weeks, defects in the test and positive groups were more extensively filled with irregular fibrous tissue, whereas the negative group showed irregular defect shapes with surrounding soft tissue redness (Figure 2D–F). By 12 weeks, the test group displayed fully regenerated, cartilage-like tissue that was well-integrated with the surrounding cartilage (Figure 2G). The positive group also showed tissue regeneration at the defect site, but the tissue appeared overgrown (Figure 2H). In contrast, the negative group exhibited incomplete tissue regeneration, with red, irregular areas persisting at the defect site (Figure 2I).

### 3.2. Histological Observations

**Figure 3:**
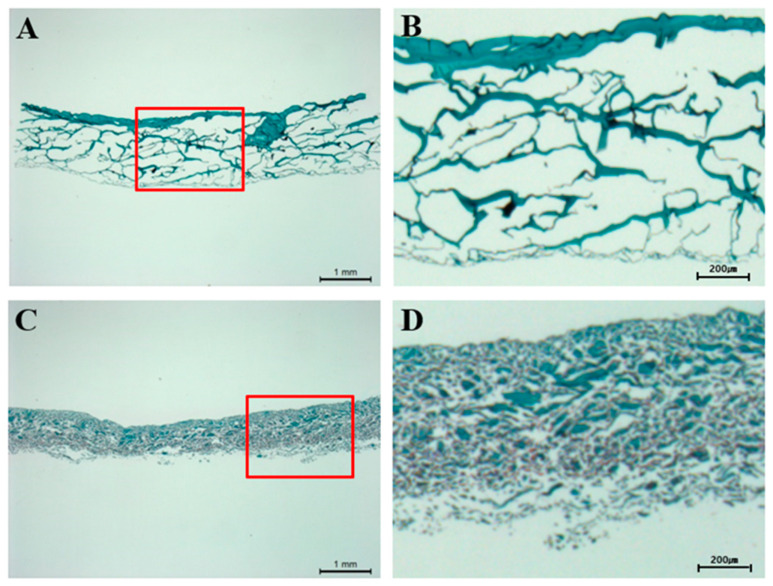
Histological analysis of scaffolds for pore size and pattern. Bilayer-atelocollagen scaffold (RegenPatch), Safranin O stain: (**A**) Scale bar = 1 mm, (**B**) Scale bar = 200 µm. Collagen scaffold (Chondro-Gide), Safranin O stain: (**C**) Scale bar 1 mm, (**D**) Scale bar = 200 µm. The panels on the right show magnified images of the red-boxed regions.

To evaluate and compare the morphological characteristics of the scaffolds, sections were stained with Safranin O. In the test group, the scaffolds exhibited a homogeneous, porous structure with an uneven pattern (Figure 3A,B). In contrast, the positive group showed scaffolds with a denser structure (Figure 3C,D).

**Figure 4:**
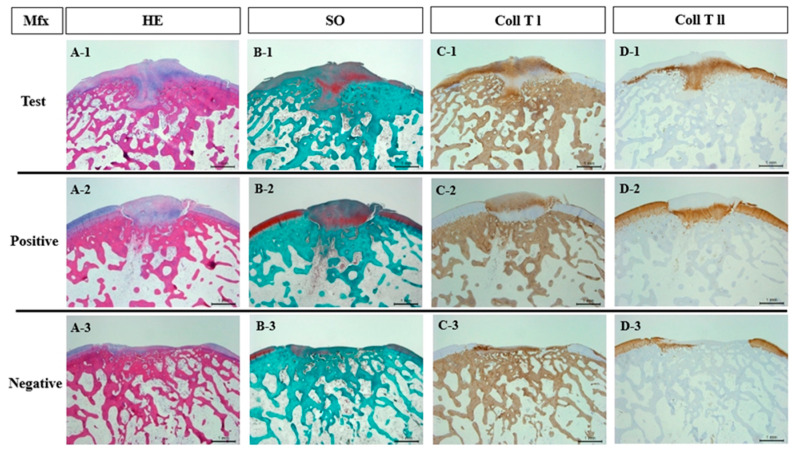
Histological and immunohistochemical evaluations of articular cartilage surface re-merging with bilayer-atelocollagen scaffolds at 12 weeks (12.5×). (**A**) HE: hematoxylin and eosin staining, (**B**) SO: safranin O staining, (**C**) Coll T I: type I collagen immunohistochemical staining, and (**D**) Coll T II: type II collagen immunohistochemical staining. The numbers correspond to the experimental groups: (**A-1**–**D-1**) rabbits at 12 weeks post--bilayer atelocollagen scaffold implantation (Test group); (**A-2**–**D-2**) rabbits at 12 weeks post-collagen scaffold implantation (Positive group); (**A-3**–**D-3**) rabbits at 12 weeks without treatment (Negative group). MFx: Microfracture. Scale bars = 1 mm.

Histological and immunohistochemical evaluations were performed at 3-, 6-, and 12-weeks post-implantation to assess tissue repair at the defect sites. Glycosaminoglycan presence was confirmed in all groups through Safranin O staining (Figure 4(B-1–B-3)). By 12 weeks, both the test and positive groups exhibited complete integration of regenerated tissue at the defect site (Figure 4(A-1,A-2)), with the test group demonstrating characteristics typical of hyaline-like cartilage. In contrast, the negative group displayed an unrepaired, damaged surface layer, with exposed cells and disrupted tissue integrity (Figure 4(A-3)). Immunohistochemical analysis confirmed that the regenerated cartilage in the test and positive groups predominantly consisted of type I and type II collagen (Figure 4(C-1,C-2,D-1,D-2)). However, in the negative group, no reparative tissue was observed, as the defect remained incompletely regenerated (Figure 4(C-3,D-3)).

**Figure 5:**
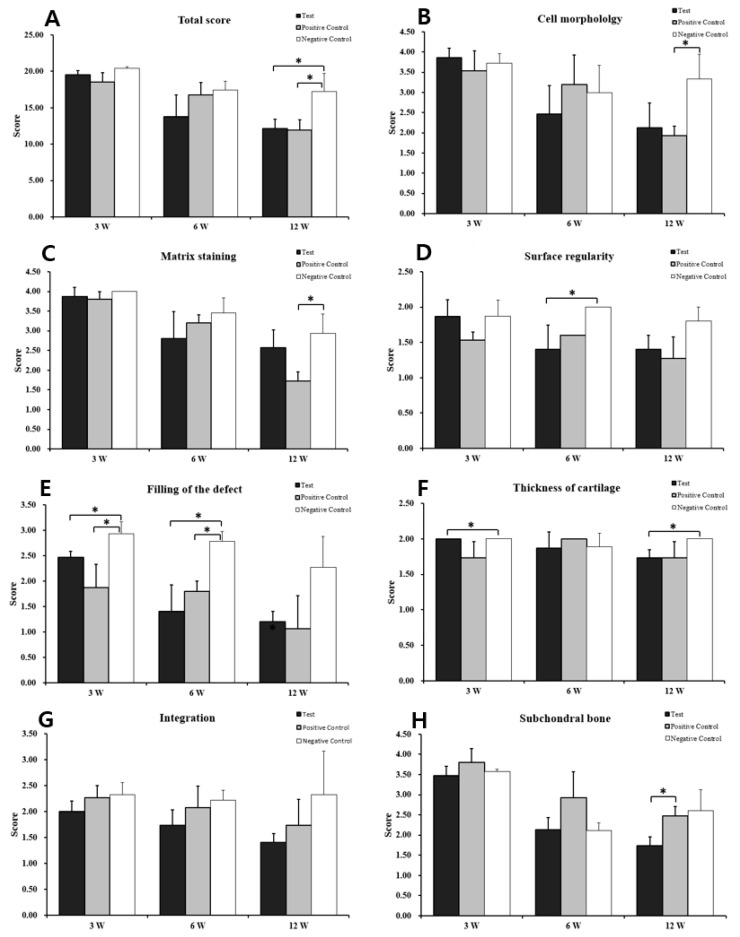
Histological evaluation scores of the regenerated cartilage. (**A**): total score; (**B**): cell morphology; (**C**): matrix staining; (**D**): surface regularity; (**E**): filling of the defect; (**F**): cartilage thickness; (**G**): integration of donor with host; (**H**): replacement of the subchondral bone. MFx: Microfracture. The total scores of the test group at 3-, 6-, and 12-weeks post-surgery were significantly higher than those of the positive group. (Student’s *t*-test *: *p* < 0.05).

The quality of regenerated tissue in each group was evaluated using the modified O’Driscoll scoring system (Table 2). At 12 weeks post-transplantation, the total scores were 12.16 ± 1.27 for the test group, 11.93 ± 1.45 for the positive group, and 17.26 ± 2.47 for the negative group. Statistical analysis revealed significant differences between the test group and the negative group (*p* = 0.033) as well as between the positive group and the negative group (*p* = 0.032) (Figure 5).

## 4. Discussion

Collagen is a primary component of the ECM in human tissues and plays a crucial role in tissue and organ development due to its excellent biocompatibility [27]. However, direct implantation of collagen can trigger an immune response as the body may recognize it as an antigen. To mitigate this, telopeptide-removed collagen (atelocollagen) is utilized to minimize immunogenic reactions [28,29]. Due to its reduced antigenicity, atelocollagen is widely applied in cartilage tissue regeneration research.

Unlike other tissues, cartilage has limited self-healing capacity due to its avascular nature and the restricted migration of chondrocytes following injury [30,31]. Moreover, once damaged, the cartilage border becomes susceptible to mechanical stress, leading to progressive wear and defect expansion. MFx is a widely used bone marrow stimulation technique for treating full-thickness articular cartilage damage, particularly in the knee and tarsal joints [32]. However, the regenerated tissue primarily consists of fibrocartilage, which lacks the biomechanical properties of hyaline cartilage, potentially leading to symptom deterioration over time. To overcome this limitation, a collagen-based scaffold is implanted following MFx to enhance bone marrow-derived stem cell migration into the defect site, thereby promoting hyaline-like cartilage formation [11,12,14,33,34]. In the previous study, we compared and evaluated the effectiveness of invented monolayer atelocollagen scaffolds to the Chondro-Gide and the negative control in Mfx and the autologous bone marrow concentrate procedure. We found that the result was better when a monolayer of atelocollagen scaffold was applied in either the Mfx or BMAC procedure for the treatment of joint defects.

In this study, the commercially available Chondro-Gide and a newly developed bilayer atelocollagen scaffold were used to treat cartilage defects following MFx, and their effectiveness was compared. The results demonstrated that the bilayer atelocollagen scaffold provided superior defect repair at 12 weeks post-treatment compared to MFx alone. This enhanced healing effect may be attributed to the scaffold’s structural design. When only MFx was performed, the released bone marrow cells were likely washed away by synovial fluid, limiting their retention at the defect site. In contrast, the bilayer scaffold facilitated cell retention and tissue regeneration. The upper layer served as a protective barrier, shielding the surgical site from external influences, while the lower porous layer confined the bone marrow-derived cells, preventing their loss and thereby enhancing the healing process.

Histological analysis revealed that both the bilayer scaffold and the positive control group achieved significantly higher scores than the negative control group at week 12 (Figure 4, Table 2). This suggests that the collagen membrane effectively creates a therapeutic environment within the defect site by concentrating MSCs and bone marrow-derived growth factors, thereby enhancing cartilage regeneration. Additionally, the bilayer scaffold and positive control group exhibited comparable histological outcomes, likely due to both being collagen-based products with similar intended applications. However, in contrast to the positive group, the test group displayed a less dense structure (Figure 3), which may be advantageous for cartilage regeneration by allowing better bone marrow infiltration. Given its comparable efficacy, the bilayer atelocollagen scaffold could serve as a new treatment option for cartilage repair, potentially offering greater accessibility compared to currently available alternatives. While this study primarily focused on histological staining to assess tissue regeneration, future research should incorporate biomechanical assessments or tensile strength tests on regenerated cartilage in animal models to provide a more comprehensive evaluation.

## 5. Conclusions

In conclusion, this animal model study evaluated the efficacy of bilayer atelocollagen scaffolds for the treatment of osteochondral defects. Given the limited regenerative capacity of damaged cartilage, implanting a scaffold to enhance tissue repair and restore cartilage function may offer a more effective solution compared to MFx alone. Our findings indicate that bilayer atelocollagen scaffold implantation significantly promoted articular cartilage regeneration, as evidenced by macroscopic, morphological, and histological analyses. This approach facilitated the formation of hyaline-like cartilage, suggesting its potential clinical utility. Overall, these results support the use of bilayer atelocollagen scaffolds in combination with MFx as a promising strategy for osteochondral defect repair.

## Figures and Tables

**Table 1:** Histological grading scale for cartilage defects.

Category	Points
Cell morphology	
Hyaline cartilage	0
Mostly hyaline cartilage	1
Mostly fibrocartilage	2
Mostly non-cartilage	3
Non-cartilage only	4
Matrix-staining	
Normal	0
Slightly reduced	1
Moderately reduced	2
Markedly reduced	3
None	4
Surface regularity	
Smooth (>3/4)	0
Slight disruption	1
Severe disruption	2
Filling of the defect	
≥111%	
91–110%	0
76–90%	1
51–75%	2
26–50%	3
≥25%	4
Thickness of cartilage	
>2/3	0
1/3–2/3	1
<1/3	2
Integration of donor with host adjacent cartilage	
Normal continuity and integration	0
Decreased cellularity	1
Gap (lack of continuity) on one side	2
Gap (lack of continuity) on one side	3
Percentage replacement of subchondral bone	
90–100%	0
75–89%	1
50–74%	2
25–49%	3
0–24%	4
Total score	23

This table was modified from the scale described by O’Driscoll et al., Pineda et al., and Wakitani et al. [25,26,27,28].

**Table 2:** Histological scoring result for the cartilage defects.

Time After Implantation		Group (n = 4)		
		Test	Positive	Negative
MFx	3 W	19.55 ± 0.58	18.53 ± 1.29	20.43 ± 0.21
	6 W	13.80 ± 2.99	16.80 ± 1.64	17.45 ± 1.17
	12 W	12.16 ± 1.27 *	11.93 ± 1.45 *	17.26 ± 2.47

Results are presented as means ± standard deviations (SD). MFx: microfracture; W: weeks; Test: bilayer-atelocollagen scaffold implant group; Positive: positive group (collagen scaffold implant); Negative: negative group (only MFx treatment). * Statistical difference compared to the negative group (*p* < 0.05).

## Data Availability

Data supporting the results of this study can be obtained from the corresponding author upon reasonable request.

## Abbreviations

The following abbreviations are used in this manuscript:

MFx	Microfracture
ECM	Extracellular matrix
MSC	Mesenchymal stem cells
ANOVA	Analysis of variance
